# Tracking physical activity in different settings from late childhood to early adulthood in Germany: the MoMo longitudinal study

**DOI:** 10.1186/s12889-015-1731-4

**Published:** 2015-04-17

**Authors:** Annette Rauner, Darko Jekauc, Filip Mess, Steffen Schmidt, Alexander Woll

**Affiliations:** Department of Sports and Sports Science, Karlsruhe Institute of Technology, Engler-Bunte Ring 15 (Building 40.40), 76131 Karlsruhe, Germany; Department of Sports Sciences, Humboldt-Universität zu Berlin, Philippstraße 13 (Building 11), 10111 Berlin, Germany; Institute of Health Sciences, University of Education Schwäbisch Gmünd, Oberbettringer Straße 200, 73525 Schwäbisch Gmünd, Germany

**Keywords:** Physical activity, MoMo, Tracking, Adolescents, Youth, Settings

## Abstract

**Background:**

Regular physical activity is important for remaining healthy. Most studies on the association between active child- and adulthood were based on small non-representative populations. The purpose of the study was to quantify tracking of leisure-time PA (in and outside sports clubs) for 6 years from adolescence into young adulthood in a representative sample in Germany.

**Methods:**

This study was a subsample of the “Motorik-Modul (MoMo) Longitudinal Study” (baseline: 2003–2006, wave 1: 2009–2012). Representative longitudinal physical activity data of N = 947 adolescents were included and collected using the MoMo-physical activity questionnaire (MoMo-PAQ). Stability of different physical activity indices was measured using Spearman’s rank-order correlations and ANOVA with repeated measurement with age, sex and socio-economic status (SES) as determinants.

**Results:**

While mean leisure-time physical activity outside sports clubs (LTPA) (F_1,397_ = 7.9, df = 1; p < .01), sports club physical activity (SCPA) (F_387_ = 4.8, df = 1; p < .05) and overall physical activity (OPA) (F_1,441_ = 7.7, df = 1; p < .01) changed significantly over time, no changes in overall sports index (OS index) (F_371_ = 3.2, df = 1; p > .05) were observed. Low tracking correlations were found for different physical activity indices (LTPA: r = .094, p < .05; SCPA: r = .248 p = <.05; OPA: r = .211 p < .05; OS index: r = .266 p < .05). Results by sex, age and SES were inconsistent. Analyses of agreement showed different results for determinants and settings.

**Conclusion:**

The results of this representative study were comparable to previous studies and showed significant but low stability. Possible reasons for low stability coefficients are a relatively long timespan between both measurement points and potential effects of the reliability of subjective assessment methods. The results confirm that physical activity is a fluctuating variable. Future studies should examine the determinants of tracking and change in physical activity.

## Background

Although the health benefits of physical activity are well documented [1], these benefits require continuous and regular participation in physical activities. A physically active lifestyle during childhood and adolescence presumably carries over into adulthood [2] implying that physical activity interventions during childhood and adolescence would be an appropriate strategy for improving physical activity levels during adulthood. Moreover, an active lifestyle during childhood and adolescence is an important factor in preventing diseases in adulthood [3-5]. Especially because obesity, physically inactive lifestyle and poor cardiorespiratory fitness in childhood may increase the risk of health problems later in life as stated by Pahkala et al. [6], an active lifestyle should start early in life [2]. Therefore, it is important to understand these carry-over or ‘tracking’ effects of physical activity from childhood through adulthood.

Twisk et al. [7] have defined the term ‘tracking’ as the stability of a certain variable over time or the predictability of a measurement early in life for the value of the same variable later in life. Usually, tracking is expressed by test-retest-correlations representing normative stability. Normative stability will be high when individuals remain at the same relative position within the sample distribution. Mean-stability (means) and homogeneity of variances provide additional information for interpreting test-retest-correlations. Telama et al. [8] revealed in their review that the tracking of physical activity is mostly low (males: r = .15 to .44; females: r = .09 to .34) from young age to adulthood where some studies showed no significant correlations. Subsequently, five studies [9-13] from Estonia, USA, Finland, Brazil, Australia, Belgium, Scotland and the Netherlands used intervals between measurements from 3 to 21 years to analyse mostly the normative and mean stability. The results of these studies were inconsistent with a tendency to low tracking correlations. Studies published after 2009 showed weak correlation coefficients for normative stability for overall populations (overall: r = −.08 to .35) [11,12,13] with slightly higher correlation coefficients for girls (r = −.07 to .39) than for boys (r = .05 to .31) [9-12]. In addition, (in)active adolescents had higher odds ratios for growing into (in)active young adults [9,12,13]. Most previous studies used questionnaires to assess total physical activity and did not distinguish between type, frequency, intensity and setting of physical activity. Some studies [11,14] used also objective measures to assess frequency, intensity or steps of physical activity. However, a comparison between studies using objective and subjective measurement methods is difficult because of different reliability. Moreover, comparisons between studies are difficult because of the large variability in study population size, intervals between measurements and lumped analyses of physical activity. Hence, to date the tracking of specific aspects of physical activity especially in representative samples in European countries is largely unknown.

Therefore, the purpose of the study was to quantify tracking of leisure-time physical activity (in and outside sports clubs) for 6 years from adolescence into young adulthood in Germany.

## Methods

### Study design and participants

KiGGS is a longitudinal and for German children and adolescents nationally representative study [15] conducted by the Robert Koch-institute in Berlin and approved by the Federal Office for Data Protection and by the ethics committee of the Charité University Hospital. In this study, nationwide representative data on health status of children and adolescents are collected and the development of health issues, health behaviour and health risks are monitored in a core survey and several modules [16]. The “Motorik-Modul” (MoMo) Longitudinal Study is one of these modules [15,17] and aims to analyse the development of physical fitness and physical activity, and the impact of physical fitness and physical activity on mental and physical health [18]. The MoMo Longitudinal Study was approved by the ethics committee of University of Konstanz and has been performed according to the Declaration of Helsinki. The MoMo participants were recruited from the KiGGS sample. The sampling procedure was based on a three-step process developed in cooperation with the Centre for Survey Research and Methodology (ZUMA, Mannheim, Germany) [15]. Based on an appropriate computation, first the study sample points (N = 167) were determined. At the second stage, an age-stratified sample of randomly selected children and adolescents was drawn from local population registries for the “KiGGS Study” with a total of 28,400 participants aged 0 to 17 years [15,19]. From these 28,400 participants, 17,641 youths took part in the “KiGGS Study” (response rate: 62.1%) [20]. In a third stage, the Robert Koch-institute has drawn the representative subsample aged between 4 and 17 years, (N = 7,866) for the MoMo Study in consultation with ZUMA [17]. 4,529 participants took part in the MoMo Study (response rate: 57.6%). To ensure the representativeness, weighting procedures were used [20].

In this study, we used a subsample of the MoMo Longitudinal Study. Prior to study participation, each parent and participant gave informed written consent. From 2003 to 2006, data of 4,529 children and adolescents aged between 4 and 17 years were collected in a nationwide representative sample (study baseline, t_0_). From 2009 to 2012, the first survey wave (t_1_) was conducted. Overall, cross-sectional data of 4,529 and longitudinal data of 2,842 children, adolescents and young adults, aged between 4 to 23 years were surveyed in the first wave. Data collection was conducted year-round without any interruptions over a longer period. Because the participants gave information in which months they participated in sports, the impact of seasonality could be excluded from the indices. In addition, the order of data collection on the 167 sample points and the time of year (season) were similar at wave 1 and baseline. Hence, an impact of seasonality can be precluded [21].

Detailed information on the study design has been previously published [18]. In this study, only longitudinal data for participants aged 11 to 17 years at baseline were included in the analysis (N_overall_ = 947; N_boys_ = 447; N_girls_ = 500). MoMo longitudinal data are available upon request (alexander.woll@kit.edu).

### Outcome measure

Physical activity was measured using the MoMo Physical Activity Questionnaire (MoMo-PAQ) for adolescents [22], which assesses physical activity in different settings (sports clubs, leisure-time, school, daily activities and overall physical activity). Beside frequency, duration and intensity, the MoMo-PAQ captured data on the type of physical activity in and outside sports clubs. Up to four different physical activities could be mentioned. For the analyses of agreement all information at t_0_ and t_1_ were matched, proofing if at both measurement points the same information were given. The MoMo-PAQ has satisfying and similar reliability and validity as other questionnaires for measuring physical activity in adolescents [22]. The reliability of assessing physical activity on the index level for one-week inter-test interval (ICC = intraclass correlation) was between r_ICC_ = .60 (p < .01) and r_ICC_ = .74 (p < .01) [22]. The ICC for overall physical activity was r = .74 (p < .01), for sports club physical activity r_ICC_ = .64 (p < .01) and for leisure-time physical activity r_ICC_ = .69 (p < .01). The validity (compared to Actigraph GT1M and the Previous Day Physical Activity Recall) was r = .24 (p < .01) and r = .43 (p < .01) for overall activity, r = .35 (p < .01) and r = .55 (p < .01) for sports club physical activity, and r = .10 and r = .32 (p < .01) for leisure-time physical activity, respectively [22].

In this study, we only considered sports club and leisure-time activities because in Germany physical activity of adolescents and young adults largely takes place in organised sports clubs. In addition, both aspects were assessed using the same instrument for both age groups at both sampling points.

*Sports club physical activity* (SCPA) was assessed by four items: type of club sports activity, duration (minutes per session) and frequency (times per week) of each club sports activity, and time of the year (month) of each club sports activity. These items were combined in a club sports activity index reflecting active minutes per week at sports clubs:

$$ \mathrm{Club}\ \mathrm{s}\mathrm{ports}\ \mathrm{index} = \left(\mathrm{duration}*\mathrm{frequency}*\mathrm{number}\ \mathrm{of}\ \mathrm{month}\right)/\left(12\ \mathrm{month}\mathrm{s}\right) $$

*Leisure-time physical activity* (LTPA) was assessed by three items: type of leisure-time physical activity, duration (minutes per week) of each leisure-time physical activity, and time of the year (month) of each leisure-time physical activity. These items were combined in the leisure-time physical activity index reflecting active minutes per week at leisure time:

$$ \mathrm{Leisure}\hbox{-} \mathrm{time}\ \mathrm{physical}\ \mathrm{activity}\ \mathrm{index} = \left(\mathrm{duration}*\mathrm{number}\ \mathrm{of}\ \mathrm{months}\right)/\left(12\ \mathrm{months}\right) $$

The *overall sports index* (OS index) was calculated by adding club sports activity and leisure-time activity index.

*Overall physical activity* (OPA) was assessed using a two-item questionnaire [23] capturing information on numbers of days during the last seven days (Item 1) and during a typical week (Item 2) of moderate physical activity of at least 60 minutes per day (not considering physical education classes). The mean of these two items combines to a scale reflecting the days per week with moderate physical activity of one hour or more according to international recommendations.

In order to estimate the change and stability of the compliance with physical activity recommendations two groups were formed (fulfilled/not fulfilled). The recommendation claims that children and adolescents aged between 5 and 17 years should perform at least 60 minutes of moderate to vigorous physical activity (of 5 to 8 metabolic equivalent of task) [24].

### Sociodemographic predictors

Sex, age and socioeconomic status (SES) were included as determinants in the analysis. Two age groups were defined for each measurement point: t_0_: 11 to 13 years (young) and 14 to 17 years (old); t_1_: 17 to 19 years (young) and 20 to 23 years (old). Socioeconomic status was assessed by a parent questionnaire asking parental educational and professional status and total income of the family household [25] and categorized into three categories low, middle and high [26].

### Statistical analyses

All statistical tests were conducted using IBM SPSS 21 (IBM Corporation, Armonk, NY). The study sample was described using descriptive analyses. The tracking of physical activity between baseline and wave 1 was analysed in different ways. First, Spearman’s rank-correlations were calculated to track physical activity of the participants. Second, analyses of variance with repeated measurement were used to examine the mean stability (means). Fisher’s Z transformation was used to compare the Spearman’s rank-order correlations between the different indices. Kappa values were analysed for categorical variables to detect agreement between both measurement points. Outliers were detected using three standard deviations of the physical active participants and excluded from further analyses. 110 cases were identified with data for club sports activity and leisure-time physical activity. Only participants with sports club membership at t_0_ and t_1_ were considered for sports club activity. The level of significance was set a priori to α = .05 for all statistical tests.

#### Missing data

Dealing with missing data is very important in empirical studies because missing data often lead to bias in parametric rating or to small sample sizes [27]. Methods for dealing with *unit-nonresponse* and *item-nonresponse* were used to accommodate for these circumstances*.* Unit-nonresponse was treated by creating a weighting procedure to account for potential bias in outcome variables caused by selective unit-nonresponse (drop-out bias) [19]. In a first step, initial design weights for the baseline sample were defined using information on the probability of selection of the measurement point and the participant within the measurement point. Subsequent data stratification ensured representativeness to the target population (German children and adolescents aged 4–17 years) regarding sex, age, region, migration background and education level. Based on these initial weights, weighted logistic regressions were performed to predict the probability of participation in wave 1. In the process, baseline data of wave 1 responders and nonresponders were used to estimate differences between wave 1 responders and the baseline sample. All longitudinal participants were assigned an individual weight according to the inverse of their probability of participation (inverse probability weighting). Expected differences between wave 1 responders and nonresponders in different variables of interest were identified, and these differences were eliminated by the weighting procedure.

For item nonresponse, the Little’s missing completely at random test [28] revealed that data was missing in a systematic way (χ^2^ = 1,091, df = 581, p < .001) on item level among participants suggesting that deletion procedures would yield biased estimates [29]. The amount of missing data due to item-nonresponse was low and ranged from 0.3% and 7.6% for all variables. Regression imputations were used to treat missing data due to item-non-response.

## Results

### Stability of means

#### Leisure-time physical activity (minutes per week)

Overall LTPA changed significantly from baseline to wave 1 (F_1,397_ = 7.9, df = 1, p = .005; Tables 1 and 2). The change in LTPA over time was greater for the young group than for the old group (F_1,397_ = 10.5, df = 1, p = .001). The young group had significantly lower LTPA at t_1_ than at t_0_ (F_123_ = 6.5, df = 1, p = .012) while in the old group LPTA remained nearly unchanged (F_1,346_ = 0.0, df = 1, p = .890). A significant interaction in LPTA between age, sex and SES was observed (F_1,397_ = 5.8, df = 2, p = .003).

**Table 1:** **Mean (1 standard deviation) patient characteristics**

		**LTPA (min./week)**	**SCPA (min./week)**	**OPA (days/week)**	**OS index (min./week)**
**Indices – t**_**0**_					
**Overall**		74.1 ± 98.0	218.6 ± 122.8	3.47 ± 1.82	292.7 ± 164.5
		N = 924	N = 296	N = 947	N = 932
**Sex**	boys	81.3 ± 107.0	231.1 ± 124.2	3.78 ± 1.80	312.4 ± 171.0
		N = 438	N = 168	N = 447	N = 438
	girls	67.3 ± 88.1	200.5 ± 118.6	3.15 ± 1.78	267.8 ± 151.2
		N = 496	N = 128	N = 500	N = 494
**Age**	young group	86.4 ± 104.5	112.6 ± 112.8	3.82 ± 1.75	199.0 ± 145.3
		N = 116	N = 55	N = 117	N = 117
	old group	72.9 ± 97.3	107.9 ± 135.0	3.43 ± 1.82	180.0 ± 179.6
		N = 818	N = 241	N = 830	N = 815
**SES**	low	64.5 ± 101.4	189.3 ± 107.1	3.29 ± 1.91	253.8 ± 134.6
		N = 185	N = 39	N = 186	N = 184
	middle	81.9 ± 100.6	224.6 ± 120.7	3.70 ± 1.76	306.5 ± 170.6
		N = 485	N = 166	N = 494	N = 483
	high	69.6 ± 89.8	222.0 ± 133.1	3.15 ± 1.78	291.6 ± 158.6
		N = 262	N = 91	N = 265	N = 263
**Indices – t**_**1**_					
**Overall**		74.1 ± 93.5	222.2 ± 133.1	3.31 ± 1.86	296.3 ± 163.2
**Sex**	boys	82.6 ± 102.1	250.5 ± 132.9	3.48 ± 1.85	333.1 ± 165.3
	girls	64.2 ± 83.5	181.0 ± 122.6	3.15 ± 1.87	245.2 ± 147.2
**Age**	young group	58.6 ± 87.2	134.4 ± 168.0	3.20 ± 1.79	193.0 ± 170.2
	old group	74.6 ± 93.9	72.3 ± 5.4	3.32 ± 1.87	146.9 ± 162.3
**SES**	low	64.2 ± 96.9	230.0 ± 109.3	3.22 ± 1.83	294.2 ± 146.4
	middle	73.3 ± 89.4	231.5 ± 132.2	3.30 ± 1.88	304.8 ± 159.5
	high	82.2 ± 97.0	199.0 ± 144.5	3.38 ± 1.88	281.2 ± 177.9
**Indices – t**_**1**_ **-t**_**0**_					
**Overall**		−0.03 ± 128.46	−3.57 ± 155.65	0.16 ± 2.30*	−3.6 ± 192.2*
**Sex**	boys	−1.34 ± 141.59	−19.37 ± 161.68	0.30 ± 2.31*	−20.7 ± 206.4*
	girls	3.05 ± 114.70	19.43 ± 143.84	0.00 ± 2.28	22.6 ± 167.7*
**Age**	young group	27.89 ± 139.01*	−21.84 ± 140.89	0.62 ± 2.10*	6.0 ± 183.0
	old group	−1.67 ± 127.16	35.67 ± 154.33	0.11 ± 2.32	33.1 ± 192.9*
**SES**	low	0.27 ± 138.53	−40.70 ± 115.00*	0.07 ± 2.12	−40.4 ± 176.7*
	middle	8.62 ± 125.98	−6.90 ± 151.05	0.40 ± 2.40*	1.7 ± 186.8
	high	−13.75 ± 124.12	22.96 ± 178.53	−0.23 ± 2.25*	10.4 ± 202.3*

(*p < .05).

SES **–** socioeconomic status; LTPA – leisure-time PA; SCPA – sport club PA; OPA – overall PA.

OS index – overall sports index. Only subjects with data for baseline and wave 1 were included in the analysis, and hence the number of subjects at t_0_ and t_1_ are the same.

**Table 2:** **Results of ANOVA with repeated measurements**

**Index**	**Source**	**Type III sum of squares**	**df**	**Mean square**	**F**	**p**
LTPA (min./week)	time	64716.3	1	64716.3	7.9	.005
	time*sex	12211.3	1	12211.3	1.5	.223
	time*age	86628.4	1	86628.4	10.5	.001
	time*SES	13236.2	2	6618.1	.8	.447
	time*sex*age	23309.3	1	23309.3	2.8	.092
	time*sex*SES	42984.8	2	21492.4	2.6	.074
	time*age*SES	40840.3	2	20420.2	2.5	.084
	time*sex*age*SES	94827.2	2	47413.6	5.8	.003
	error(time)	11340256.6	1379	8223.5		
SCPA (min./week)	time	55058.5	1	55058.5	4.8	.030
	time*sex	25927.2	1	25927.2	2.2	.135
	time*age	87432.4	1	87432.4	7.6	.006
	time*SES	10686.4	2	5343.2	.5	.630
	time*sex*age	322.8	1	322.8	0.0	.867
	time*sex*SES	42454.7	2	21227.4	1.8	.160
	time*age*SES	73267.2	2	36633.6	3.2	.043
	time*sex*age*SES	56027.5	2	28013.7	2.4	.090
	error(time)	4465186.7	387	11538.0		
OPA (days/week)	time	20.1	1	20.1	7.7	.005
	time*sex	0.0	1	0.0	0.0	.974
	time*age	15.3	1	15.3	5.9	.015
	time*SES	42.8	2	21.4	8.2	<.001
	time*sex*age	2.1	1	2.1	0.8	.373
	time*sex*SES	8.9	2	4.5	1.7	.180
	time*age*SES	12.4	2	6.2	2.4	.093
	time*sex*age*SES	8.4	2	4.2	1.6	.199
	error(time)	3747.1	1441	2.6		
OS index (min./week)	time	58825.1	1	58825.1	3.2	.074
	time*sex	2907.8	1	2907.8	0.2	.691
	time*age	22768.8	1	22768.8	1.2	.266
	time*SES	17939.7	2	8969.9	0.5	.613
	time*sex*age	17924.6	1	17924.6	1.0	.323
	time*sex*SES	1710.1	2	855.1	0.0	.954
	time*age*SES	26708.8	2	13354.4	0.7	.483
	time*sex*age*SES	1047.5	2	523.8	0.0	.972
	error(time)	6798404.1	371	18324.5		

(*p < .05).

LTPA – leisure-time PA; SCPA – sport club PA; OPA – overall PA; OS index – overall sports index.

#### Sport club physical activity (minutes per week)

Mean SCPA increased significantly from baseline to wave 1 (F_387_ = 4.8, df = 1, p = .030; Tables 1 and 2). Age was the only determinant that significantly affected the change in SCPA over time (F_387_ = 6.3, df = 1, p < .012). In addition, a significant interaction in SCPA between time, age, SES was observed (F_387_ = 3.2, df = 2, p = .043). While the young group showed greater SCPA over the time independent of SES, subjects with high SES showed lower SCPA and those with low or middle SES showed higher SCPA over time. SCPA decreased by 21.8 minutes per week from t_0_ to t_1_ in the young group (F_55_ = 17.4, df = 1, p < .001).

#### Overall physical activity (days per week)

Mean OPA decreased significantly from baseline to wave 1 (F_1,441_ = 7.7, df = 1, p = .005; Tables 1 and 2). The analyses revealed a significant age (F_1,441_ = 5.9, df = 1, p = .015) and SES effect (F_1,441_ = 8.2, df = 2, p < .001). The young group (F_126_ = 10.3, df = 1, p = .002) and the old group (F_1,346_ = 3.7, df = 1, p = .054) had significant lower OPA at t_1_ than at t_0_. The post hoc test (Scheffé) showed that participants with a middle SES had a significantly higher OPA than those with a high SES A significant interaction between SES and time was found. According to the post hoc test (Scheffé), participants with a middle SES greater change in OPA than those with a high SES (middle difference: 0.219, p = .05, CI = .000/.438). Participants with a middle or high SES had a significant decrease in OPA from t_0_ to t_1_. OPA increased significantly in boys but not in girls (Table 1).

#### Overall sports index (minutes per week)

Mean overall OS index did not change significantly from baseline to wave 1 (F_371_ = 3.2, df = 1, p = .074; Tables 1 and 2). No significant interactions between OS index and sociodemographic predictors were detected.

### Tracking

#### Leisure-time physical activity

The Spearman’s rank-order correlation of LTPA ranged from r = −.015 to r = .184 representing a weak correlation for tracking physical activity from adolescence to young adulthood for LTPA independent of the determinants (Table 3). The overall correlation coefficients was r = .094 (p < .001). Girls had significant correlation coefficients (N = 488, r = .109, p = .003) in LTPA while the tracking of LTPA in boys was not significant (N = 427, r = .072, p = .061). However, the correlation coefficients did not differ between boys and girls (z = 0.56, p = .576). Spearman’s rank-order correlations were significant only in the old group (old group: N = 800, r = .102, p < .001; young group: N = 115, r = .063, p = .494). The correlation coefficients did not differ between the young and old groups (z = 0.39, p = .697).

**Table 3:** **Spearman’s rank-order correlation**

		**LTPA (min./week)**	**SCPA (min./week)**	**OPA (days/week)**	**OS index (min./week)**
**Overall**		.094*	.248*	.211*	.266*
**Sex**	boys	.072	.214*	.198*	.200*
	girls	.109*	.239*	.201*	.332*
**Age**	young group	.063	.244	.274*	.242
	old group	.102*	.254*	.208*	.275*
**SES**	low	-.015	.416*	.371*	.269*
	middle	.075	.228*	.115*	.301*
	high	.184*	.194*	.224*	.234*

(*p < .05).

LTPA – leisure-time PA; SCPA – sport club PA; OPA – overall PA; OS index – overall sports index.

Spearman’s rank-order correlations were significant only for participants with a high SES (low: N = 182, r = −.015, p = .785; middle: N = 472, r = .075, p = .053; high: N = 259, r = .184, p < .001; Table 3). The correlation coefficients did not differ between the low and middle SES groups (z = 1.03, p = .303), and the middle and high SES groups (z = 1.44, p = .150). The coefficients differed significantly between the low and high SES groups (z = 2.07, p = .039). Overall, tracking of LTPA was weak for all analysed groups.

#### Sport club physical activity

The Spearman’s rank-order correlation of SCPA ranged from r = .194 to r = .416 representing a higher stability than LTPA (Table 3). Weak to moderate tracking coefficients of SCPA from adolescence to young adulthood were found. The overall tracking value was r = .248 (p < .001). SCPA correlated significantly in girls (N = 126, r = .239, p = .002) and in boys (N = 164, r = .214, p = .001). The correlation coefficients did not differ between boys and girls (z = 0.22, p = .826). Spearman’s rank-order correlations were significant only in the old group (old group: N = 347, r = .254, p < .001; young group: N = 52, r = .244, p = .082) and did not differ significantly between age groups (z = 0.07, p = .944). Spearman’s rank-order correlations were significant in all SES groups (low: N = 59, r = −.416, p = .001; middle: N = 225, r = .228, p = .001; high: N = 115, r = .194, p = .037). The correlation coefficients did not differ significantly between the low and middle SES groups (z = 1.14, p = .254), between the low and high SES groups (z = 1.24, p = .215) and between the middle and high SES groups (z = 0.27, p = .787).

#### Overall physical activity

OPA showed weak to moderate stability correlations (r = .115 to r = .371) and weakly tracked from t_0_ to t_1_ (r = .211, p < .001; Table 3). OPA had significant correlation coefficients in girls (N = 500, r = .201, p < .001) and in boys (N = 477, r = .198, p < .001). The correlation coefficients did not differ between boys and girls (z = 0.05, p = .960). Spearman’s rank-order correlations were significant in the young (N = 117, r = .274, p = .002) and in the old groups (N = 830, r = .208, p < .001) with no significant differences in stability coefficients (z = 0.69, p = .490). Spearman’s rank-order correlations were significant in all SES groups (low: N = 183, r = .371, p < .001; middle: N = 477, r = .115, p = .002; high: N = 262, r = .224, p < .001). The stability coefficient of OPA was significantly higher in the low SES group than in the middle SES group (z = 3.55, p < .001). The correlation coefficients did not differ between the low and high SES groups (z = 1.67, p = .095) and between the middle and high SES groups (z = 1.28, p = .201).

#### Overall sports index

The OS index showed moderate stability correlations (r = .200 to r = .332) with low to moderate strength of relation from t_0_ to t_1_ (r = .266, p < .001; Table 3). Correlation coefficients for the OS index were significant in girls (N = 161, r = .332, p < .001) and in boys (N = 222, r = .200, p = .003). The correlation coefficients did not differ significantly between boys and girls (z = 1.36, p = .174). Spearman’s rank-order correlations were weak to moderate but not significant in the young (N = 52, r = .242, p = .083) and the old groups (N = 331, r = .275, p < .001). Rank-order correlation coefficients did not differ significantly between age groups (z = 0.23, p = .818). Tracking values were significant in all SES groups (low: N = 59, r = .269, p = .04; middle: N = 210, r = .301, p < .001; high: N = 119, r = .234, p = .012). No significant differences between the different classes of SES were found: middle and low SES (z = 0.23, p = .818), middle and high SES (z = 0.27, p = .535), low and high SES (z = 0.23, p = .818).

Correlation coefficients were significantly lower for LTPA (r = .094) than those for OS index (r = .266; z = 4.71, p < .001), OPA (r = .211; z = 3.19, p = .001) and SCPA (r = .248, z = 2.79, p = .005). OS index was more stable than the OPA (z = 4.25, p < .001) and SCPA (z = 2.11, p = .030), No differences were found for the stability between OPA and SCPA (z = 0.69, p = .490), OPA and OS index (z = 1.56, p = .119) and between OPA and SCPA (z = 0.69, p = .490).

### Changes in physical activity groups

The descriptive analyses showed a negative trend, independently of setting. More persons shifted after six years from being active to inactive, independently of setting (LTPA: active-inactive: 54%, inactive-active: 46%; SCPA: member in sport club-not member in sport club: 77.1%, not member in sport club-member in sport club: 22.9%; OS index: active-inactive: 64.3%, inactive-active: 35.7%; OPA: fulfilled-unfulfilled: 58.9%, unfulfilled-fulfilled: 41.1%).

For all settings, the analyses of agreement revealed a poor to fair strength kappa coefficient. The agreement between t_0_ and t_1_ of active and inactive, respectively, was 8.9% (k = .089, p = .001) for LTPA; 36.1% (k = .361, p < .001) for SCPA (member/not member); 17.6% (k = .176, p < .001) for OS index and 7.2% (k = .072, p = .005) for OPA (recommendations fulfilled/not fulfilled).

### Changes in type of physical activity

SCPA showed small agreement between both measurement points (22.9%, k = .229, p < .001), whereas no agreement between t_0_ and t_1_ in LTPA was observed (k = .000, p > .05).

No Gender differences were found in the analyses of agreement. For girls and boys, no agreement between both measurement points was found for SCPA (k = .000, p > .05) and for LTPA (k = .000, p > .05). The analyses of agreement differed between age groups. No agreement of type in SCPA was found in the young age group (k = .000, p > .05) but the old age group showed an agreement of 19.8% (k = .198, p < .001). For LTPA no agreement were found for both age groups (k = .000, p > .05). The analyses of agreement did not differ between SES classes. No agreement of type in SCPA for low SES was found (k = .000, p > .05), for middle SES 31.2% (k = .312, p < .001) and for high SES 12.7% (k = .127, p < .001). In contrast, LTPA showed no agreement (k = .000, p > .05) in all SES classes.

## Discussion

The aim of this nationwide representative study was to quantify tracking of leisure-time physical activity (in and outside sports clubs) for 6 years from adolescence into young adulthood in Germany. The results for overall mean stability were inconsistent. While significant changes over time in mean LTPA, SCPA and OPA were observed, OS index did not changed significantly over time. These results suggest that mean OS index is stable over 6 years from adolescence to young adulthood. Significant interactions between time and the determinants age, sex and SES were found for physical activity in all settings. Of the analysed determinants, age had the strongest influence on stability of physical activity in different settings.

While other studies showed higher correlation coefficients for boys [30], sex differences in stability of physical activity in different settings in our study showed inconsistent results. The changes in mean SCPA and mean OPA in boys were higher than in those in girls, but the changes in mean LTPA and PA in girls were higher than those in boys. Consequently, a general statement regarding tracking physical activity in all settings for girls and boys cannot be made. However, separate analyses by sex showed stable mean values in LTPA and SCPA for boys and in LTPA, SCPA and OPA for girls. Possible reasons for changes in physical activity in different settings for girls and for boys include increasingly demanding school requirements [31], the transition from school to other forms of education and the associated limited availability of time [32], relocation or changing personal interests during puberty. Similarly, Gordon-Larson and colleagues determined that young adults are in a period of changes [33]. Other studies showed that—especially for girls—the commitment in physical activity decreased the more demanding the physical activity is [34-36]. Consequently, girls invested less time per week at baseline resulting in smaller overall changes to wave 1. In addition, the support for physical activity from peers [37-39] and parents [37-39] are the strongest factors for identification with physical activities [40]. In case of relocation this support can disappear and evoke changes in physical activity.

Changes in LTPA, SCPA and OS index did not differ between the three SES groups. However, the middle SES group showed significantly greater changes in OPA than the high SES group. These results indicate that the mean physical activity remains stable over the timespan of 6 years independent of social classes.

Stability coefficients did not differ between girls and boys supporting results reported by Herman et al. [41]. In contrast, Telama et al. [4,42] reported lower stability of LTPA, SCPA and OPA in girls than in boys. A possible reason for these discrepancies is that girls may have other interests in their leisure time (such as fashion or music) during puberty than boys [32,43]. While for boys the competition may be important [44], for girls the sociability played an important role [45]. Other studies observed that girls rather engage in physical activities if a same-sex friend joins them in their activity [44]. In our study, tracking coefficients were low for boys and girls. These results confirm previous reports of low tracking coefficients for LTPA, SCPA and OPA [4,42,46,47]. Hence physical activity in adolescence and young adulthood appears to be a volatile behaviour exposed to many destabilising factors.

Rank-order coefficients did not differ between age groups. These results do not correspond to the results of the study by Telama et al. [4] who reported highest correlations in the oldest cohort. However, in that study the young group was very small (N = 55) compared to the old group (N = 830). Hence, it is possible that this age difference does not fully represent true differences between groups, and hence these results should be interpreted with caution.

Differences in stability coefficients between SES groups were observed for LTPA and OPA but not for SCPA and OS indices. As expected, LTPA tracked better in the high SES groups than in the low SES group. In contrast, OPA was more stable in the low SES group than in the middle SES group. Several factors may explain these differences. It is possible that parents of the young age group with a high SES have greater (financial) resources [48] allowing their children to participate in activities other than sports such as playing music requiring increasing time commitments as the children age. In contrast, greater financial resources enable to participate in physical activities [48,49]. Another possible reason is the fact that in this study a large number of the study population relocated after finishing high school requiring reorganising physical and sports activities in the new environment.

In Germany, training in sports clubs is scheduled regularly each week on the same day and time with only minor variations between years [4]. Hence, it was to expect that SCPA is more stable than physical activity outside of sports clubs [30]. However, the results of this study showed that the stability of physical activity did not differ between settings independently of sex, age and SES. Hence, it appears that adolescents who are physically active will not change the setting (sports club versus outside of sports clubs) of their activity as they grow into young adults. Moreover, the peer environment and a relationship of trust to the trainer may explain these results.

In general, comparing our results with those of other studies is difficult because the timespan between sampling points and the age range of study population vary largely between studies [3,8,42,50]. Moreover, most studies only analysed the stability of overall physical activity. Nonetheless, our results are in agreement with those of other studies [7,51,52] with similar study population and intervals. For instance, Twisk et al. [7] found a stability coefficient of r = .340 while other studies reported only rarely significant correlation coefficients but rather significant predictions or tendencies [51,52].

Results of the analyses of agreement for the type of physical activities are inconsistent. While the SCPA showed no agreement for girls and boys, the middle social class showed the highest agreement in SCPA. In addition, only the old age group showed an agreement between both measurement points which could be caused by the small number of participants in the young age group. LTPA showed no agreement for all determinants and age groups.

The correlation coefficients in our study were low which may have been related to the long timespan of 6 years between baseline and wave 1. Indeed, previous studies showed that the longer the interval is the smaller are the stability values. For instance, Basterfield et al. [53] reported rank-order correlations between baseline and wave 1 (2 years interval) of r = .540. The correlation coefficients in our study ranged from .094 to .416. Correlation coefficients in a study with a 22-year interval by Basterfield et al. [53] were not significant. These combined results confirm that physical activity tracks better the shorter the timespan between measurements [30]. The strength of correlations also depends on the reliability of assessing subjective information [4]. Specifically, tracking correlations not only entail stability of the measurement but also its reliability. Therefore, low reliability of physical activity measures is another potential reason for the reported low test-retest-correlations. Therefore, future studies should consider using objective measures for assessing stability of physical activity from adolescence to young adulthood.

### Strength and limitations

In this study, we measured physical activity in different settings in a large and representative study sample with a large age range. The focus of our study was to analyse “physical activity”. In Germany, socially conditioned, this physical activity happened mostly in sports clubs, why this aspect played an important role in the analyses. However, overall physical activity was also analysed. Because of the size of the sample, data were collected using a questionnaire that only captures subjective data. Questionnaires tend to overestimate physical activity, and the study sample tends to have difficulties to appreciate the extent (in frequency, duration and intensity) of physical activity [54]. This could lead to diverse information between the measurement points resulting in weak stability over time. Moreover, it is possible that estimated missing data over- or underestimated physical activity values. Finally, the satisfactory reliability may have resulted in lower stability coefficients thereby underestimating stability. Because of the higher reliability of objective measures, an increase of the test-retest correlation would be expected if physical activity would be gather with e.g. accelerometers.

## Conclusions

In this representative study, we report on the stability of physical activity in different settings. The results showed that physical activity in different settings is not stable over time and confirmed that physical activity is a fluctuating variable. The poor stability of physical activity over time emphasize the necessity for strategies aiming at making physical activity programs more attractive to adolescents and increasing physical activity in young adults [47].

## Abbreviations

MoMo: Motorik Modul
MoMo-PAQ: MoMo-physical activity questionnaire
ICC: Intraclass correlation
LTPA: Leisure-time physical activity
SCPA: Sport club physical activity
OPA: Overall physical activity
OS index: Overall sports index
SES: Socioeconomic status

## References

[1] Li J, Siegrist J (2012). Physical activity and risk of cardiovascular disease–a meta-analysis of prospective cohort studies. Int J Environ Res Public Health.

[2] Hallal PC, Victora CG, Azevedo MR, Wells JC (2006). Adolescent physical activity and health: a systematic review. Sports Med.

[3] Lefevre J, Philippaerts RM, Delvaux K, Thomis M, Vanreusel B, Eynde BV (2000). Daily physical activity and physical fitness from adolescence to adulthood: A longitudinal study. Am J Hum Biol.

[4] Telama R, Leskinen E, Yang X (1996). Stability of habitual physical activity and sport participation: a longitudinal tracking study. Scand J Med Sci Sport.

[5] Twisk JW, Kemper HC, van Mechelen W (2002). Prediction of cardiovascular disease risk factors later in life by physical activity and physical fitness in youth: general comments and conclusions. IJSM.

[6] Pahkala K, Hernelahti M, Heinonen O, Raittinen P, Hakanen M, Lagström H (2012). Body mass index, fitness and physical activity from childhood through adolescence. Br J Sports Med.

[7] Twisk JW, Kemper HC, van Mechelen W (2000). Tracking of activity and fitness and the relationship with cardiovascular disease risk factors. Med Sci Sports Exerc.

[8] Telama R (2009). Tracking of physical activity from childhood to adulthood: a review. Obes Facts.

[9] Dumith SC, Gigante DP, Domingues MR, Hallal PC, Menezes AMB, Kohl HW (2012). A longitudinal evaluation of physical activity in Brazilian adolescents: tracking, change and predictors. Pediatr Exerc Sci.

[10] Swaminathan S, Selvam S, Thomas T, Kurpad AV, Vaz M (2011). Longitudinal trends in physical activity patterns in selected urban south Indian school children. Indian J Med Res.

[11] Kelly LA, Reilly JJ, Jackson DM, Montgomery C, Grant S, Paton JY (2007). Tracking physical activity and sedentary behavior in young children. Pediatr Exerc Sci.

[12] Jose KA, Blizzard L, Dwyer T, McKercher C, Venn AJ (2011). Childhood and adolescent predictors of leisure time physical activity during the transition from adolescence to adulthood: a population based cohort study. Int J Behav Nutr Phys Act.

[13] Cleland V, Dwyer T, Venn A (2012). Which domains of childhood physical activity predict physical activity in adulthood? A 20-year prospective tracking study. Br J Sports Med.

[14] Cleland VJ, Ball K, Magnussen C, Dwyer T, Venn A (2009). Socioeconomic position and the tracking of physical activity and cardiorespiratory fitness from childhood to adulthood. Am J Epidemiol.

[15] Kurth BM, Kamtsiuris P, Holling H, Schlaud M, Dolle R, Ellert U (2008). The challenge of comprehensively mapping children’s health in a nation-wide health survey: design of the German KiGGS-Study. BMC Public Health.

[16] Spengler S, Mess F, Schmocker E, Woll A (2014). Longitudinal associations of health-related behavior patterns in adolescence with change of weight status and self-rated health over a period of 6 years: results of the MoMo longitudinal study. BMC Pediatr.

[17] Woll A, Kurth BM, Opper E, Worth A, Bos K (2011). The ‘Motorik-Modul’ (MoMo): physical fitness and physical activity in German children and adolescents. Eur J Pediatr.

[18] Wagner MO, Bos K, Jekauc D, Karger C, Mewes N, Oberger J. et al. Cohort Profile: The Motorik-Modul Longitudinal Study: physical fitness and physical activity as determinants of health development in German children and adolescents. Int J Epidemiol. 2013. doi:10.1093/ije/dyt098 10.1093/ije/dyt098 23847291

[19] Kamtsiuris P, Lange M, Schaffrath Rosario A (2007). [The german health interview and examination survey for children and adolescents (KiGGS): sample design, response and nonresponse analysis]. Bundesgesundheitsblatt.

[20] Jekauc D, Reimers AK, Wagner MO, Woll A (2012). Prevalence and socio-demographic correlates of the compliance with the physical activity guidelines in children and adolescents in Germany. BMC Public Health.

[21] Lange M, Butschalowsky HG, Jentsch F, Kuhnert R, Schaffrath Rosario A, Schlaud M (2014). The first KiGGS follow-up (KiGGS Wave 1). Study conduct, sample design, and response. Bundesgesundheitsblatt.

[22] Jekauc D, Wagner MO, Kahlert D, Woll A (2013). Reliability and validity of MoMo-Physical-Activity-Questionnaire for Adolescents (MoMo-AFB). Diagnostica.

[23] Prochaska JJ, Sallis JF, Long B (2001). A physical activity screening measure for use with adolescents in primary care. Arch Pediatr Adolesc Med.

[24] World Health Organisation. Global Recommendations on Physical activity for Health. 2013. http://www.who.int/dietphysicalactivity/factsheet_recommendations/en/.

[25] Lampert T, Schenk L, Stolzenberg H (2002). Konzeptualisierung und Operationalisierung sozialer Ungleichheit im Kinder- und Jugendgesundheitssurvey. [Conceptualisation and Operationalisation of Social Inequality in the National Health Interview and Examination Survey for Children and Adolescents]. Gesundheitswesen.

[26] Winkler J, Stolzenberg H (1999). [Social Status Scaling in the German National Health Interview and Examination Survey]. Gesundheitswesen.

[27] Jekauc D, Völkle M, Lämmle L, Woll A (2012). Fehlende Werte in sportwissenschaftlichen Untersuchungen [Missing data in examination in sports science]. Sportwissenschaft.

[28] Little RJA (1988). A Test of Missing Completely at Random for Multivariate Data with Missing Values. J Am Stat Assoc.

[29] Schaffer JL, Graham JW (2002). Missing data: our view of the state of the art. Psychol Methods.

[30] Malina RM (1996). Tracking of physical activity and physical fitness across the lifespan. Res Q Exerc Sport.

[31] Garcia AW, Broda MA, Frenn M, Coviak C, Pender NJ, Ronis DL (1995). Gender and developmental differences in exercise beliefs among youth and prediction of their exercise behavior. J Sch Health.

[32] Tappe MK, Duda JL, Ehrnwald PM (1989). Perceived barriers to exercise among adolescents. J Sch Health.

[33] Gordon-Larsen P, Nelson MC, Popkin BM (2004). Longitudinal physical activity and sedentary behavior trends: adolescence to adulthood. Am J Prev Med.

[34] Fuchs R, Powell KE, Semmer NK, Dwyer JH, Lippert P, Hoffmeister H (1988). Patterns of physical activity among German adolescents: the Berlin-Bremen Study. Prev Med.

[35] van Mechelen W, Twisk JW, Post GB, Snel J, Kemper HC (2000). Physical activity of young people: the Amsterdam Longitudinal Growth and Health Study. Med Sci Sports Exerc.

[36] Trost SG, Pate RR, Sallis JF, Freedson PS, Taylor WC, Dowda M (2002). Age and gender differences in objectively measured physical activity in youth. Med Sci Sports Exerc.

[37] McGuire MT, Neumark-Sztainer DR, Story M (2002). Correlates of time spent in physical activity and television watching in a multi-racial sample of adolescents. Pediatr Exerc Sci.

[38] Zakarian JM, Hovell MF, Hofstetter CR, Sallis JF, Keating KJ (1994). Correlates of vigorous exercise in a predominantly low SES and minority high school population. Prev Med.

[39] Anderssen N, Wold B (1992). Parental and peer influences on leisure-time physical activity in young adolescents. Res Q Exerc Sport.

[40] Neumark-Sztainer D, Story M, Hannan PJ, Tharp T, Rex J (2003). Factors associated with changes in physical activity: a cohort study of inactive adolescent girls. Arch Pediatr Adolesc Med.

[41] Herman KM, Craig CL, Gauvin L, Katzmarzyk PT (2009). Tracking of obesity and physical activity from childhood to adulthood: the Physical Activity Longitudinal Study. Int J Pediatr Obes.

[42] Telama R, Yang X, Viikari J, Valimaki I, Wanne O, Raitakari O (2005). Physical activity from childhood to adulthood: a 21-year tracking study. Am J Prev Med.

[43] Kientzler A (1999). Fifth- and seventh-grade girls’decisions about participation in physical activity. Elem School J.

[44] Flintoff A, Scraton S (2001). Stepping into Active Leisure? Young women’s perceptions of active lifestyles and their experiences of school physical education. Sport Educ Soc.

[45] Hargreaves J (1994). Sporting Females: critical issues in the history and sociology of women’s sports.

[46] Anderssen N, Wold B, Torsheim T (2005). Tracking of physical activity in adolescence. Res Q Exerc Sport.

[47] Janz KF, Dawson JD, Mahoney LT (2000). Tracking physical fitness and physical activity from childhood to adolescence: the muscatine study. Med Sci Sports Exerc.

[48] Dagkas S, Stathi A (2007). Exploring social and environmental factors affecting adolescents’ participation in physical activity. Eur Phys Educ Rev.

[49] Roberts K, Cavill N, Hancock C, Rutter H (2013). Social and economic inequalities in diet and physical activity.

[50] Matton L, Thomis M, Wijndaele K, Duvigneaud N, Beunen G, Claessens AL (2006). Tracking of physical fitness and physical activity from youth to adulthood in females. Med Sci Sports Exerc.

[51] Tammelin T, Nayha S, Laitinen J, Rintamaki H, Jarvelin MR (2003). Physical activity and social status in adolescence as predictors of physical inactivity in adulthood. Prev Med.

[52] Engström L, Oja P, Telama R (1991). Exercise adherence in sport for all from youth to adulthood. Sport for all.

[53] Basterfield L, Adamson AJ, Frary JK, Parkinson KN, Pearce MS, Reilly JJ (2011). Longitudinal study of physical activity and sedentary behavior in children. Pediatrics.

[54] Lohman TG, Ring K, Pfeiffer K, Camhi S, Arredondo E, Pratt C (2008). Relationships among fitness, body composition, and physical activity. Med Sci Sports Exerc.

