# Correction to: Tracking physical activity in different settings from late childhood to early adulthood in Germany: the MoMo longitudinal study

**DOI:** 10.1186/s12889-018-5111-8

**Published:** 2018-02-05

**Authors:** Annette Rauner, Darko Jekauc, Filip Mess, Steffen Schmidt, Alexander Woll

**Affiliations:** 1 Department of Sports and Sports Science, Karlsruhe Institute of Technology, Engler-Bunte Ring 15 (Building 40.40), 76131 Karlsruhe, Germany; 2 Department of Sports Sciences, Humboldt-Universität zu Berlin, Philippstraße 13 (Building 11), 10111 Berlin, Germany; 3 Institute of Health Sciences, University of Education Schwäbisch Gmünd, Oberbettringer Straße 200, 73525 Schwäbisch Gmünd, Germany

## Correction

After publication of the article [1], it has been brought to our attention that the values cited under “indices t1-t0” have been reversed and should be displayed in the opposite order. The corrected Table 1 would appear as follows

**Table 1:** Mean (1 standard deviation) patient characteristics (*p < .05)

		LTPA (min./week)	SCPA (min./week)	OPA (days/week)	OS index (min./week)
indices – t_0_					
overall		74.1 ± 98.0 N=924	218.6 ± 122.8 N=296	3.47 ± 1.82 N=947	292.7 ± 164.5 N=932
sex	boys	81.3 ± 107.0 N=438	231.1 ± 124.2 N=168	3.78 ± 1.80 N=447	312.4 ± 171.0 N=438
	girls	67.3 ± 88.1 N=496	200.5 ± 118.6 N=128	3.15 ± 1.78 N=500	267.8 ± 151.2 N=494
age	young group	86.4 ± 104.5 N=477	193.6 ± 101.6 N=181	3.82 ± 1.75 N=477	280.0 ± 145.3 N=117
	old group	72.9 ± 97.3 N=469	227.1 ± 126.8 N=130	3.43 ± 1.82 N=469	300.0 ± 179.6 N=815
SES	low	64.5 ± 101.4 N=185	189.3 ± 107.1 N=39	3.29 ± 1.91 N=186	253.8 ± 134.6 N=184
	middle	81.9 ± 100.6 N=485	224.6 ± 120.7 N=166	3.70 ± 1.76 N=494	306.5 ± 170.6 N=483
	high	69.6 ± 89.8 N=262	222.0 ± 133.1 N=91	3.15 ± 1.78 N=265	291.6 ± 158.6 N=263
indices – t_1_					
overall		74.1 ± 93.5	222.2 ± 133.1	3.31 ± 1.86	296.3 ± 163.2
sex	boys	82.6 ± 102.1	250.5 ± 132.9	3.48 ± 1.85	333.1 ± 165.3
	girls	64.2 ± 83.5	181.0 ± 122.6	3.15 ± 1.87	245.2 ± 147.2
age	young group	58.6 ± 87.2	232.9 ± 190.0	3.20 ± 1.79	291.5 ± 170.2
	old group	74.6 ± 93.9	228.9 ± 138.8	3.32 ± 1.87	303.5 ± 162.3
SES	low	64.2 ± 96.9	230.0 ± 109.3	3.22 ± 1.83	294.2 ± 146.4
	middle	73.3 ± 89.4	231.5 ± 132.2	3.30 ± 1.88	304.8 ± 159.5
	high	82.2 ± 97.0	199.0 ± 144.5	3.38 ± 1.88	281.2 ± 177.9
indices – t_1_-t_0_					
overall		0.0 ± 128.5	3.6 ± 155.7	-0.2 ± 2.3*	3.6 ± 192.2*
sex	boys	1.3 ± 141.6	19.4 ± 161.7*	-0.3 ± 2.3*	20.7 ± 206.4*
	girls	-3.1 ± 114.7	-19.4 ± 143.8*	0.0 ± 2.3	-22.6 ± 167.7*
age	young group	-27.9 ± 139.0*	39.3 ± 140.9*	-0.6 ± 2.1*	10.5 ± 183.0*
	old group	1.7 ± 127.2	1.8 ± 154.3	-0.1 ± 2.3	3.5 ± 192.9*
SES	low	-0.3 ± 138.5	40.7 ± 115.0*	-0.1 ± 2.1	40.4 ± 176.7*
	middle	-8.6 ± 126.0	6.0 ± 151.1	-0.4 ± 2.4*	-1.7 ± 186.8
	high	13.8 ± 124.1*	-23.0 ± 178.5*	0.2 ± 2.3*	-10.4 ± 202.3*

SES **–** socioeconomic status; LTPA – leisure-time PA; SCPA – sport club PA; OPA – overall PA;

OS index – overall sports index

Furthermore, the last sentence of Sport club physical activity (minutes per week) that reads “SCPA decreased by 39.3 min per week from t0 to t1 in the young group (F177 = 15.4, df = 1, *p* < .001).” should in fact say “SCPA increased by 39.3 min per week from t0 to t1 in the young group (F177 = 15.4, df = 1, *p* < .001).”
